# Editorial: Refractive errors: public health challenges and interventions

**DOI:** 10.3389/fpubh.2023.1289173

**Published:** 2023-10-02

**Authors:** Carla Lanca, Chi Pui Pang, Andrzej Grzybowski

**Affiliations:** ^1^Escola Superior de Tecnologia da Saúde de Lisboa (ESTeSL), Instituto Politécnico de Lisboa, Lisboa, Portugal; ^2^Comprehensive Health Research Center (CHRC), Escola Nacional de Saúde Pública, Universidade Nova de Lisboa, Lisboa, Portugal; ^3^Department of Ophthalmology and Visual Sciences, The Chinese University of Hong Kong, Hong Kong, China; ^4^Hong Kong Hub of Paediatric Excellence, The Chinese University of Hong Kong, Hong Kong, China; ^5^Joint Shantou International Eye Center, Shantou University, Shantou, China; ^6^Department of Ophthalmology, University of Warmia and Mazury, Olsztyn, Poland; ^7^Institute for Research in Ophthalmology, Foundation for Ophthalmology Development, Poznań, Poland

**Keywords:** myopia, public health, refractive errors, prevention, health promotion

Uncorrected refractive errors are a leading cause of visual impairment and blindness across many countries. Refractive errors that are not corrected during the critical period of visual system development may lead to serious conditions, such as amblyopia. The largest burden of refractive error is myopia which significantly increases the risk of blinding conditions such as myopic macular degeneration, glaucoma, and cataract. Previous studies show that vision impairment in children is associated with symptoms of depression and anxiety, and children with myopia revealed higher scores of depression compared with children having normal vision (1). Substantial impact in the economic health of individuals as well as decreased educational and employment opportunities have been associated with visual impairment and blindness in adults (2, 3). Decreased quality of life, increased risk of falls and increased risk of death have also been associated with visual impairment and blindness in older adults (4–7). Additionally, visual impairment may coexist with other health conditions, amplifying the impact of comorbidities, thereby increasing the disability risk (8). In the past few years, epidemiological research has shown that uncorrected refractive errors are a major public health issue in many parts of the world. However, more research is needed to determine the full extent of the threat posed by refractive errors, to establish effective interventions and to consolidate prevention efforts.

This Research Topic comprises 28 studies including original research articles and reviews on refractive errors, such as research trends and prevalence, risk factors, retinal biomarkers, treatment, and health promotion.

## Research trends and prevalence

Yang et al. described the global trends of research on pathologic myopia using a bibliometric analysis, showing that countries such as China, USA, and Japan have the greatest influence. Several studies on the prevalence of refractive errors show the burden on urban Asia regions. For example, Wang Y. et al. concluded that urban settings are more affected by myopia and anisometropia compared to rural areas in Dalian, China. Many studies reported increases in myopia prevalence during the COVID-19 lockdowns and research is moving to identify if those trends persisted after the pandemic. In Xuzhou, China, Zhou et al. concluded that the prevalence of myopia increased during the COVID-19 epidemic. The trends of myopia development remained stable in the post-COVID-19 epidemic period in younger children but increased for older children. In Chengdu, China, Pan et al. found significant increases on myopia and axial length (AL) that remained 1 year after the lockdown. Interestingly, there are regional differences in the prevalence of refractive errors in China. Wang W. et al. reported that the prevalence of refractive errors in children and adolescents was lower in Tibet than in Chongqing during the COVID-19 pandemic, probably due to higher outdoor time and lower time of use of digital devices in Tibet. The prevalence of myopia also varies with age. You et al. found a lower prevalence of myopia in preschool children younger than 5 years old. Myopia slightly increased from 5 to 6 years of age, which may indicate an early sign of myopia in school-age children in the Changsha children eye study. Several risk factors have been associated with myopia, and educational pressure seems to be a consistent factor across studies, namely the number of years of education and possibly the type of educational activities. The study by Liu et al. confirmed that the prevalence of myopia increased in students aged between 15 and 18 years in China from standard high schools, remaining stable among students from vocational schools where there were less academic work and more physical activities in the curriculum.

Myopia diagnosis can be challenging, mainly due to inaccuracy of measures without cycloplegia. Can myopia be accurately measured without the side effects caused by cycloplegics? Big data acquisition and machine learning technique have been utilized for prediction of spherical equivalent (SE) in school-age children. Du B. et al. found that the difference before and after cycloplegia can be effectively predicted in school-age children, providing evidence for the usefulness of machine learning in epidemiological studies of myopia in large populations.

Another refractive error that needs attention is anisometropia particularly due to its association with amblyopia. Xu et al. found a prevalence of 13% in Chinese children aged 4–17 years, which was associated with several risk factors, namely indoor near work time and outdoor activity time. According to Wu Z. et al. the prevalence of total astigmatism (28%) increased with age and astigmatism correction was effective, improving visual quality. Correction of refractive errors is indeed of paramount relevance. Latif et al. observed a significant impact in the average academic scores of Pakistanis high school children in Lahore after refractive corrections.

## Risk factors

For the past few years several research studies connected sleep with myopia. Nevertheless, significant gaps in knowledge still exist. Although Huang et al. found an association between sleep and myopia during the COVID-19 pandemic in Chinese children, the association was not significant after adjusting for other risks factors. Other environmental factors may also be associated with myopia. For example, Li et al. found that sedentary time was strongly related to the prevalence of poor vision among Chinese children and adolescents. On the other hand, Du W. et al. found associations between anthropometric parameters and refractive error in school-age children during the post-COVID-19 pandemic. These three studies show the need for further research on environmental factors to develop effective interventions to prevent myopia and to predict myopia risk and myopia progression at early stages.

## Retinal biomarkers

The search for retinal biomarkers related to refractive errors is increasing. Hui et al. assessed choroidal vascularity and choriocapillaris blood perfusion in Chinese children with anisometropic hyperopic amblyopia. They found that subfoveal and peripapillary choroidal thickness was higher in amblyopic children and correlated with shorter AL and higher SE, affecting choroidal structure and vascular density. The authors concluded that choroidal blood flow may be decreased in amblyopic eyes. Similarly, Zhu et al. found higher subfoveal choroidal thickness in high hyperopic eyes that correlated with shorter AL and higher SE, indicating decreased choroidal blood flow in amblyopic eyes. Structural changes in the retina may lead to functional changes and proper diagnosis is necessary to avoid delaying care. Chan et al. described the role of myopic titled disc and its implications on patient's quality of life and cost of treatment. The authors concluded that strategies to overcome examination errors should also be thoroughly explored.

## Treatment

New publications on the control of myopia progression continue to emerge each year. In the past few years such new information had profound effects on ophthalmology practice. Nowadays treating myopia progression is of paramount importance. Children that progress about half a dioptre per year until their teenage years are at great risk of developing high myopia and potential blinding conditions, such as macular myopic maculopathy. In a systematic review on the cost-effectiveness analysis of myopia management Agyekum et al. concluded that 0.01% atropine and corneal refractive surgery were cost-effective in the treatment of myopia. More importantly, the results of the study show that prevention of myopia progression is more cost-effective than treating pathologic myopia.

Machine learning (ML) is rapidly evolving, allowing for more accurate prediction of treatment effects. Fang et al. developed a ML-assisted model to assist eye care professionals in the management and prediction of the effect of orthokeratology.

The choice of atropine concentration has been the subject of intensive discussions over the past few years. Recent studies concluded that atropine 0.01% is not as effective as 0.05% in Asian children. According to Lanca et al. low-dose atropine 0.01% was not effective in reducing AL progression in two studies. However, treatment efficacy with low-dose atropine of 0.05% showed good efficacy. Nevertheless, studies published in this Research Topic, still find that atropine 0.01% is effective. For example, Yu et al. found that children, aged 6–13 years from Shanghai, treated with 0.01% atropine had less myopia progression during the lockdown compared with children that discontinued the treatment. However, the authors also found that the effect of atropine can be strengthened with defocus incorporated multiple segments lenses (DIMS) lenses. Wang M. et al. concluded that 0.02% and 0.01% atropine are effective in the control of myopia progression, mainly by reducing AL elongation, with no clinical effects on corneal and lens power, ocular and corneal astigmatism, anterior chamber depth or intraocular pressure compared to the control group.

Myopes have a higher risk of developing cataracts and cataract management in highly myopic eyes can be challenging. The review by Du Y. et al. described those challenges advocating for a prudent choice of the formula to calculate the intraocular lens (IOL) power. Additionally, the authors called for consideration of the necessary clinical parameters for selection of the correct IOL to achieve the best possible surgery outcomes.

Sub-Tenon's bupivacaine injection is a widely used local anesthesia technique for strabismus surgery. However, published research regarding effectiveness is not consistent. Weijuan et al. found that sub-Tenon's bupivacaine injection relieved postoperative pain, reducing the incidence of oculocardiac reflex and vomiting. In addition, the injection reduced the use of supplementary drugs in the management of strabismus surgery.

## Health promotion

Interventions to prevent myopia are essential to curb the myopia epidemic. Jiang et al. found that the combination of the health belief model and the theory of planned behavior improved parents' intentions to prevent myopia in preschool children. Wearing appropriate correction is essential for most refractive errors to avoid visual impairment. Wu L. et al. showed that providing free spectacles along with educational interventions can lead to higher compliance. According to Kodjebacheva et al. several issues remain in the promotion of the use of eyeglasses based on a study on Romani families in Bulgaria. Although the study subjects needed eyeglasses, they did not have any at pre-test of the intervention. Further interventions need to consider how to educate individuals on the importance of eye examinations. Thereby, increasing the adherence to eye examinations and use of eyeglasses may be advocated. Social media should also have an important role in health promotion educating adolescents regarding myopia. However, caution is necessary as Ming et al. found that content was of moderate-to-poor reliability and with variable quality on TikTok. Nevertheless, online videos may serve as an additional source of information if providers can ensure reliable content.

We hope that the scientific research findings in this Research Topic help scientists to find new hypotheses, thereby accelerating further innovation in refractive error's management. More importantly, the newly generated evidence combined with known evidence from reported studies may assist in the development of health and education policies to improve the health of patients with refractive errors.

## Author contributions

CL: Writing—original draft. CP: Writing—review and editing. AG: Writing—review and editing.
